# Latent classes based on clinical symptoms of military recruits with mental health issues and their clinical responses to treatment over 12 months

**DOI:** 10.1192/j.eurpsy.2024.569

**Published:** 2024-08-27

**Authors:** D.-I. Jon, J. Eo, E. H. Park, H. J. Hong

**Affiliations:** Psychiatry, Hallym University Hospital, Anyang, Korea, Republic Of

## Abstract

**Introduction:**

In South Korea, all men at the age of 18 or older are required to serve at military for a certain period as an obligation. These recruits should be able to withstand psychological stress and pressures of rapid adaptation of the unique and new environment in military. The number of military recruits facing adaptation issues has been on the rise, necessitating an evaluation for active service. In our previous study (Park et al., in press 2023), we classified the military recruits with mental issues according to latent profile analysis (LPA) and examined the treatment response during six months.

**Objectives:**

In this study, we further examined clinical characteristics over the next six months.

**Methods:**

Ninety-two participants were analyzed with LPA using MMPI-2 clinical profiles in the previous study. The three classes were identified: mild maladjustment (Class 1, n=14), neurotic depression and anxiety (Class 2, n=36), high vulnerability and hypervigilance (Class 3, n=42). At 12 months, Clinical Global Impression-Severity and Global Assessment of Functioning were assessed to test their long-term changes.

**Results:**

While Class 1 and 2 significantly improved over 6 months, Class 3 showed little or no improvement even with more medications in our previous study. During the 6-month follow-up period, 50% of Class 1, 38.9% of Class 2, and 41.5% of Class 3 were dropped. It was during this period that their level of military service was decided. Class 1 and 2 which showed marked improvement up to initial 6 months, did not demonstrate substantial further improvement during follow-up period with a considerable portion stopped visiting hospital. Subjects in Class 3, who showed little or no improvement during initial 6 months, demonstrated continued improvement in this study, although their symptoms still appeared relatively severe.

**Conclusions:**

This study suggests clinical implications for treatment plan and intervention of each subgroup classified based on MMPI-2 clinical profiles of military recruits who might show maladjustment to serve. The long-term continuous treatment for Class 3 patients will be needed, even after exemption from active duty.

**Disclosure of Interest:**

None Declared

